# Evaluation of small bowel motion and feasibility of using the peritoneal space to replace bowel loops for dose constraints during intensity-modulated radiotherapy for rectal cancer

**DOI:** 10.1186/s13014-020-01650-z

**Published:** 2020-09-01

**Authors:** Siyuan Li, Yanping Gong, Yongqiang Yang, Qi Guo, Jianjun Qian, Ye Tian

**Affiliations:** 1 Department of Radiotherapy & Oncology, Second Affiliated Hospital of Soochow University, Institute of Radiotherapy and Oncology, Soochow University, Suzhou Key Laboratory for Radiation Oncology, Suzhou, 215004 China; 2 Department of Oncology, Zhang Jia Gang First Hospital, Suzhou, 215004 China; 3 Department of Radiology, Second Affiliated Hospital of Soochow University, Suzhou, 215004 China

**Keywords:** Rectal cancer, Intensity modulated radiotherapy (IMRT), Small bowel, Bowel loops, Peritoneal space, Normal tissue complication probability (NTCP)

## Abstract

**Background:**

The goal of this study was to assess small bowel motion and explore the feasibility of using peritoneal space (PS) to replace bowel loops (BL) via the dose constraint method to spare the small bowel during intensity-modulated radiotherapy (IMRT) for rectal cancer.

**Methods:**

A total of 24 patients with rectal cancer who underwent adjuvant or neoadjuvant radiotherapy were selected. Weekly repeat CT scans from pre-treatment to the fourth week of treatment were acquired and defined as Plan, 1 W, 2 W, 3 W, and 4 W. The 4 weekly CT scans were co-registered to the Plan CT, BL and PS contours were delineated in all of the scans, an IMRT plan was designed on Plan CT using PS constraint method, and then copied to the 4 weekly CT scans. The dose-volume, normal tissue complication probability (NTCP) of the small bowel and their variations during treatment were evaluated.

**Results:**

Overall, 109 sets of CT scans from 24 patients were acquired, and 109 plans were designed and copied. The BL and PS volumes were 250.3 cc and 1339.3 cc. The V_15_ of BL and PS based plan of pre-treatment were 182.6 cc and 919.0 cc, the shift% of them were 28.9 and 11.3% during treatment (*p* = 0.000), which was less in the prone position than in the supine position (25.2% vs 32.1%, *p* = 0.000; 9.9% vs 14.9%, *p* = 0.000). The NTCP_C_ and NTCP_A_ based plan of pre-treatment were 2.0 and 59.2%, the shift% during treatment were 46.1 and 14.0% respectively. Majority of BL’s D_max_ and V_15_ were meet the safety standard during treatment using PS dose limit method except 3 times (3/109) of V_15_ and 5 times of D_max_ (5/109).

**Conclusions:**

This study indicated that small bowel motion may lead to uncertainties in its dose volume and NTCP evaluation during IMRT for rectal cancer. The BL movements were significantly greater than PS, and the prone position was significantly less than the supine position. It is feasibility of using PS to replace BL to spare the small bowel, V_15_ < 830 cc is the dose constraint standard.

## Background

Radiochemotherapy is a widely accepted treatment mode in patients with locally advanced rectal cancer. It can result in a significant reduction in the local recurrence rate by up to 30% and improve the 5-year disease-free survival rate [1–6].

Although radiochemotherapy can help cure many rectal cancer survivors, acute and chronic intestinal side effects (12–50%) such as diarrhoea, faecal incontinence, and late small bowel obstructions have attracted increasing attention because they may affect patients’ quality of life and even interrupt treatment [7–10]. Studies have shown that the irradiated small bowel volume is closely related to toxicity caused by radiotherapy, so reducing its irradiated volume is the key approach to effectively prevent and reduce toxicity [11, 12]. Although intensity-modulated radiation therapy (IMRT) reduces the risk of radiation-induced toxicity, toxicity remains a significant concern.

In 2010, the Quantitative Analysis of Normal Tissue Effects in the Clinic (QUANTEC) review provided the available dose volume data for small bowel toxicity. Acute small bowel injury has been described with a threshold dose of grade 3 or greater toxicity when 120 cc volume of individually contoured bowel loops (BLs) receive ≥15 Gy or when 195 cc of the contoured peritoneal space (PS) receives ≥45 Gy [13, 14]. These are commonly incorporated into radiotherapy protocols in clinical practise.

Contouring the PS and BL are two primary ways to evaluate the small bowel dose volume [15, 16]. However, the small bowel is always in motion and there may be uncertainties in dose volume evaluations. The characteristics of narrow high dose distribution in IMRT technology will further increase this uncertainty.

PS contouring has the advantages of accuracy, convenience, and repeatability compared with BL contouring. This volume can allow the small bowel to lie at any point during treatment and can mitigate the impact of small bowel movements. The scope of this study was to quantify the impact of small bowel movements on the dose volume and normal tissue complication probability (NTCP) estimates and feasibility of using PS replace BL for dose constraints to provide an optimised method of sparing the small bowel during IMRT for rectal cancer.

## Methods

### Patients

The ethics board of the hospital approved the present study, and all of the investigations were conducted in accordance with the relevant guidelines and regulations. From March 2014 to March 2016, 24 patients with rectal cancer who underwent adjuvant or neoadjuvant radiotherapy were selected. The patient characteristics are summarised in Table 1.

**Table 1:** Patient characteristics

Variable	*n*	%
Gender		
Male	15	62.5
Female	9	37.5
Age (y)		
Range	39–77	/
Median	58.5	/
T stage		
T2	4	16.7
T3	18	75.0
T4	2	8.3
N stage		
N0	8	33.3
N1	13	54.2
N2	3	12.5
Clinical stage		
II	10	41.7
III	14	58.3
Treatment position		
Supine	12	50.0
Prone	12	50.0
Radiotherapy		
Adjuvant	16	66.7
Neoadjuvant	8	33.3

### Planning CT

CT scans (3 mm thick slices) of the patients’ whole abdomen and pelvis were obtained with the treatment position on a Siemens Emotion-Duo CT simulator. Standard commercial immobilisation devices were applied. A carbon fibre frame and thermoplastic mask fixation (Pelvicast system, Orfit, Wijnegem, Belgium) was used. The patients were in the supine position with a pillow under their heads. Their knees and ankles were supported with vacuum cushions, and their arms resting on their chests. In the prone position, a belly board (Civco Medical Solutions, Coralville, IA, USA) was applied to allow the abdomen to extend into its aperture. The patients were instructed to empty the bladder an hour before CT simulation. Gastrografin solution (600 mL) was administered orally an hour before scanning to better visualise the small bowel for delineation. CT scans were subsequently imported into the treatment planning system (Pinnacle 9.0, Philips Radiation Oncology, Fitchburg, MA. USA) for target delineation and treatment planning design. After the plan was confirmed, the patients were treated at the Medical Synergy Accelerator (Elekta Synergy, Elekta Oncology Systems, Crawley, UK), and when treatment they were required to keep their bladder moderately filled similar to simulation. CT images were obtained and defined as 1 W, 2 W, 3 W, and 4 W, respectively, on the Friday of weeks 1–4 during treatment under the same scanning conditions. Subsequently, the 4 weekly CT scans were automatically co-registered to the Plan CT respectively based on pelvic bone anatomy, algorithm of Normalized Mutual Information in treatment planning system was used.

### Delineation of PS and BL

Per the delineation methods of small bowel from RTOG [17] and Robyn B [16], BL and PS were delineated for each patient’s group of CT images. BL was delineated along the bowel loop’s outer surface based on the contrast effect of Gastrografin solution and excluding the colon. The upper boundary was 1 cm above the superior level of the planning target volume (PTV), and the lower boundary was delineation of the small bowel until it ended. For the PS, the anterior and bilateral boundaries were the inner surface of the abdominal muscles, the posterior boundary was the vertebral body, sacrum, or sigmoid colon. The upper boundary was 1 cm above the superior PTV level. The lower boundary was parallel to the inferior sigmoid colon level. The PS included the small bowel and colon, but did not include the bladder, ovary, and uterus. A window width of 600 and window level of 40 were selected for delineation of the BL and PS and were completed by the same senior attending physician.

### Target volume definition and treatment planning design

The target volume was delineated per the RTOG and NCCN guidelines [18, 19]. The clinical target volume (CTV) included the lymphatic drainage area of the perirectal lymph nodes, presacral lymph nodes, and internal iliac lymph nodes, and some patients’ external iliac lymph nodes were included. A margin of 1 cm in the cranial-caudal direction and 0.5 cm in the anterior-posterior and lateral directions was given to the CTV to form the PTV. The prescription was 50 Gy in 25 fractions to the PTV. In the Pinnacle 9.0 treatment planning system, 7 field IMRT plans used the PS (V_15_ < 830 cc) dose constraints were designed [16]. The plans used a 6 MV X-ray CC convolution algorithm and a 0.3 cm computational grid. An Elekta Synergy accelerator and 40 pairs of MLCs were selected. Dose constraints of V_40_ < 50% and V_50_ < 5% were used for the bladder and bilateral femoral head respectively. The target dose coverage required more than 95% of the PTV covered by 100% of the prescription dose and a maximum dose (D_max_) < 54 Gy inside and outside the PTV. Subsequently, the IMRT plans from the Plan CT were copied to the 1-4 W CT images which had co-registered to the Plan CT.

### Evaluation of small bowel dose volume

The absolute irradiated volume (cc) of the small bowel was described by its volume exposed to 5–50 Gy with 5 Gy intervals. Each patient’s small bowel volume (or irradiated volume) was expressed by the mean value over their CT images. All of the patients’ small bowel volumes (or irradiated volumes) during treatment were expressed as their median volume values.

### Evaluation of small bowel motion

The shift% was used to describe the small bowel movements, and shift% = SD/mean [20]. The SD and mean were the standard deviation and mean of the small bowel volume (or irradiated volume) from all of the CT images. The variations among the patients were expressed by their median values. A larger shift% signified greater motion of the small bowel during treatment.

### NTCP prediction of small bowels

The Lyman-Kutcher-Burman (LKB) calculation module in Pinnacle 9.0 was used to predict chronic complications of the small bowel (called NTCP_C_) [21–23]. The n (volume factor), m (slope of dose response curve), and TD_50_ (mean dose of 50% complication probability) parameters were set to 0.15, 0.16, and 55 Gy, respectively [24]. The complications were defined as small bowel obstructions, perforations, or fistulas. Logistic formula NTCP = (1 + (V_50_/V)^k^)^− 1^ was used to calculate the acute toxicity of the small bowel based on its V_15_ (called NTCP_A_), where V_50_ and k were 130 cc and 1.1, respectively [25]. Each patient’s NTCP was expressed by the mean value over their all of the CT images. The NTCP of all of the patients during treatment was expressed by their median values. The shift% here was used to describe the NTCP variations during treatment, and shift% = SD/mean.

### Safety assessment of small bowel during treatment

V_15_ < 275 cc from Robyn B et al. [16] and D_max_≦54Gy were used as the criteria for safety evaluation of the small bowel during treatment. The small bowel was at risk when the value exceeded these criteria.

### Statistical analysis

SPSS 19.0 software was used for the data analysis. Sigma Plot 10.0 and Microsoft Excel 2007 were used for figure plotting. A paired sample t-test was used to compare the differences between the two groups’ data, and their correlation was analysed via Pearson’s correlation coefficient. A two-tailed value of *p* < 0.05 was considered statistically significant.

## Results

### PS and BL contours and treatment plans

**Fig. 1:**
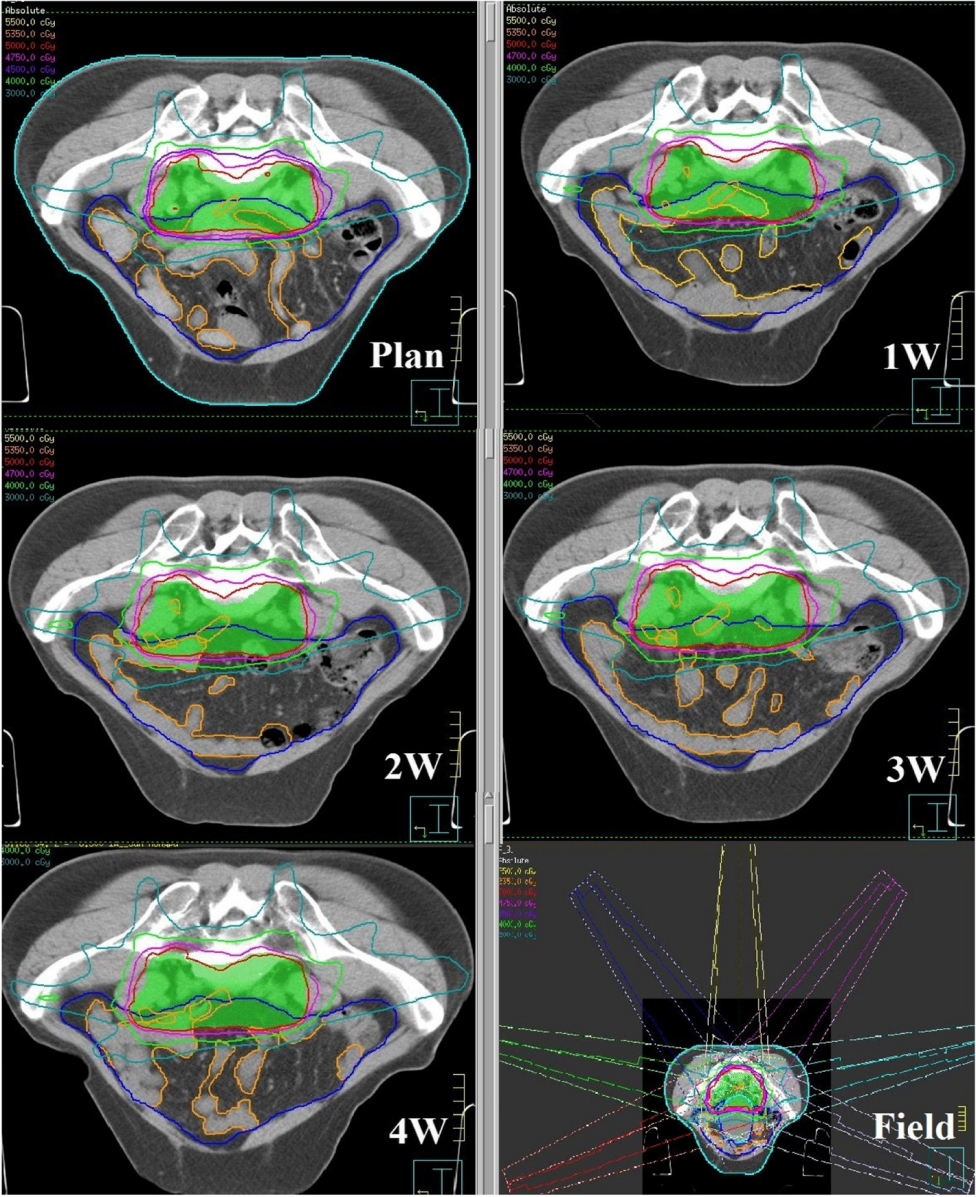
An example of a rectal cancer patient’s PS and BL contours and dose distribution based on different CT scans during treatment. The green, blue, and orange contours represent PTV, PS, and BL, respectively. The innermost and outermost dose lines are 50 Gy and 30 Gy, respectively. Picture Plan, 1 W, 2 W, 3 W, 4 W show the variations of PS and BL’s contours and dose distribution during treatment, picture Field show the radiotherapy field setup

Figure 1 shows an example of a rectal cancer patient’s PS and BL contours and dose distribution based on different CT scans during treatment. A total of 109 sets of CT images were obtained for 24 patients, including 24 sets of Plan, 2 W, and 3 W scans, 14 sets of 1 W scans, and 23 sets of 4 W scans. Overall, 218 contours containing the PS and BL were delineated for each patient. The median PS volume was 1339.3 cc (537.3–2121.7 cc) and the median BL volume was 250.3 cc (81.0–590.8 cc) in all of the patients. A total of 24 sets of IMRT plans were designed based on Plan CT (109 sets of plans obtained after the plans copied to 1-4 W CT scans). In plan of pre-treatment, the median V_15_ of the PS was 919.0 cc (493.4–1324.6 cc), and 13 sets (13/24) were V_15_ > 830 cc, all of the other dose constraints were met (the V_15_ of BL was≦275.71 cc).

### Evaluation of small bowel motion

**Fig. 2:**
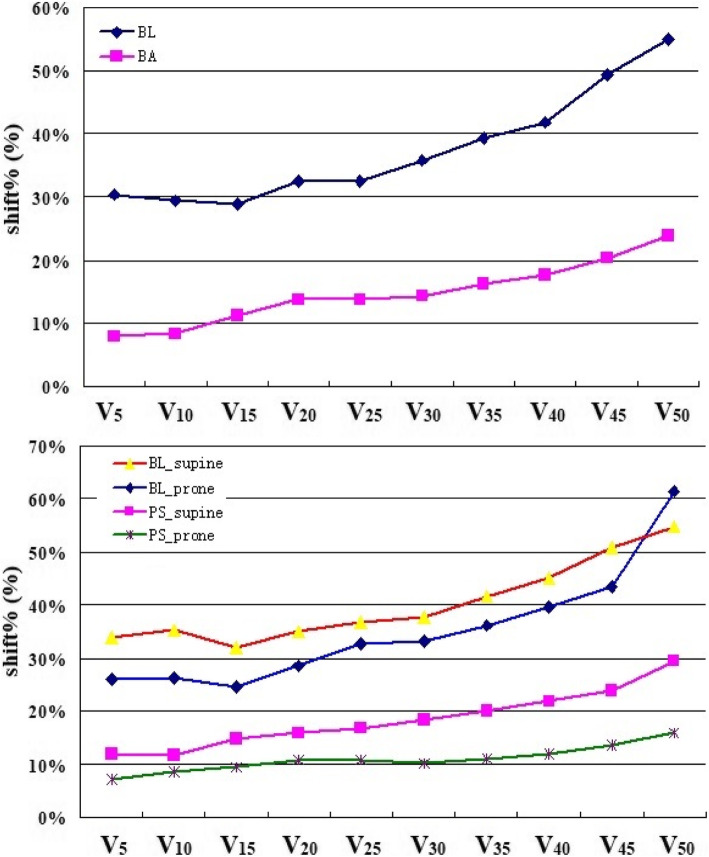
The shift% of the BL and PS’ dose-volume during treatment. The top picture show the difference of shift% between the BL and PS, dark blue and purple lines represent BL and PS respectively. The bottom picture show the difference of shift% between the supine and prone position, the red and dark blue lines represent shift% of BL in supine and prone position respectively, the purple and green lines represent shift% of PS in supine and prone position respectively

**Fig. 3:**
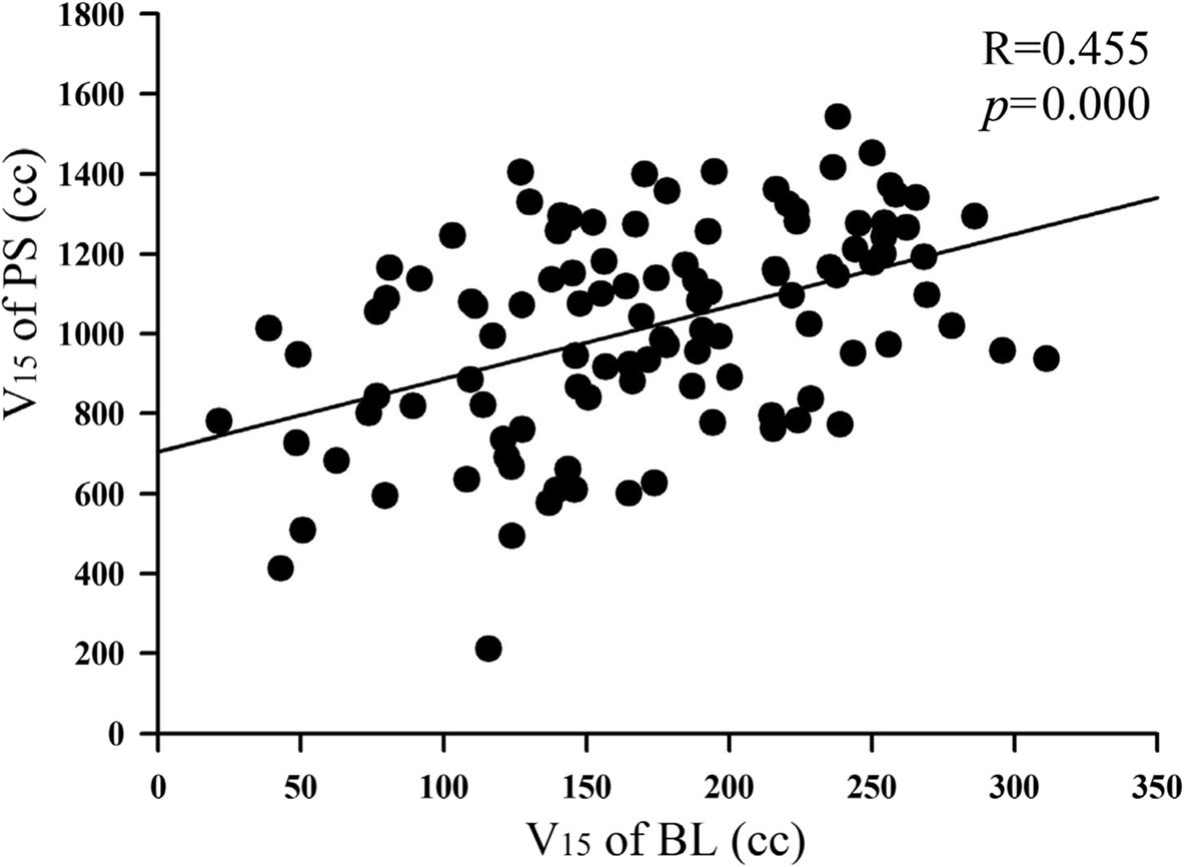
Correlation of V_15_ between the PS and BL based on all of the CT scans

The shift% of the BL and PS volumes was 28.5% (11.8–80.8%) and 9.8% (2.8–38.7%), respectively. The movement of BL was significantly larger than PS (*p* = 0.000). As shown in Fig. 2 and Table 2 and 3, the shift% of dose-volume (V_5–50_) from 28.9–55.0% in BL was significantly larger than the PS of 7.9–23.8% (top picture of Fig. 2). The shift% of the BL and PS’s V_15_ were 28.9% (4.8–72.2%) and 11.3% (3.2–42.8%) (*p* = 0.000) respectively, and the shift% of V_30_ were 35.8% (3.8–88.8%) and 14.4% (4.2–47.3%) respectively (*p* = 0.000). The shift% of the BL and PS’s V_15_ in the prone position was lower than in the supine position (25.2% vs 32.1%, *p* = 0.000; 9.9% vs 14.9%, *p* = 0.000). As shown in Fig. 3, there was a significant correlation of V_15_ between the PS and BL during tratmnt, R = 0.455, *p* = 0.000.

**Table 2:** The dose-volume and NTCP of BL and their shift% during treatment

Variable	Plan	1 W	*P*_*1*_	2 W	*P*_*2*_	3 W	*P*_*3*_	4 W	*P*_*4*_	Mean	SD	shift%
V_5_ (cc)	288.9	356.3	0.897	210.0	0.009	263.8	0.169	224.0	0.007	248.7	75.5	30.4%
V_10_ (cc)	244.0	270.2	0.902	187.4	0.012	223.8	0.441	197.1	0.015	223.4	65.8	29.4%
V_15_ (cc)	182.6	219.0	0.731	144.6	0.042	181.5	0.887	167.3	0.023	170.1	49.1	28.9%
V_20_ (cc)	152.8	173.5	0.985	120.4	0.058	166.0	0.639	125.3	0.036	139.2	45.3	32.6%
V_25_ (cc)	125.7	139.7	0.858	91.1	0.126	148.3	0.924	105.2	0.084	112.2	36.5	32.6%
V_30_ (cc)	99.9	116.1	0.611	73.1	0.277	111.1	0.641	81.0	0.171	82.3	29.5	35.8%
V_35_ (cc)	79.2	98.9	0.439	55.5	0.45	85.4	0.405	60.8	0.185	63.9	25.1	39.3%
V_40_ (cc)	61.1	72.0	0.339	42.3	0.795	65.3	0.199	48.9	0.260	50.5	21.1	41.8%
V_45_ (cc)	42.3	47.3	0.215	32.3	0.743	48.3	0.152	34.1	0.368	37.2	18.4	49.4%
V_50_ (cc)	17.8	23.2	0.088	19.2	0.131	22.1	0.028	18.5	0.539	21.6	11.9	55.0%
NTCP_A_ (%)	59.2	63.9	0.958	52.9	0.046	59.1	0.891	56.9	0.017	56.5	7.9	14.0%
NTCP_c_ (%)	2.0	2.0	0.110	3.0	0.034	4.0	0.007	3.0	0.323	2.8	1.3	46.1%

The *P*_*1*_*, P*_*2*_*, P*_*3*_*,* and *P*_*4*_ represent the comparison between the 1-4 W and Plan respectively

**Table 3:** The dose-volume and its shift% of PS during treatment

Variable	plan	1 W	*P*_*1*_	2 W	*P*_*2*_	3 W	*P*_*3*_	4 W	*P*_*4*_	Mean	SD	shift%
V_5_ (cc)	1222.1	1444.7	0.147	1319.4	0.010	1359.5	0.083	1344.9	0.334	1328.6	105.1	7.9%
V_10_ (cc)	1094.8	1319.9	0.054	1222.4	0.741	1104.6	0.921	1169.6	0.824	1217.8	101.9	8.4%
V_15_ (cc)	919.0	1091.4	0.143	1098.1	0.015	1075.1	0.081	1082.1	0.351	1000.4	113.0	11.3%
V_20_ (cc)	796.8	981.0	0.071	949.9	0.015	974.5	0.003	956.3	0.103	910.5	125.0	13.7%
V_25_ (cc)	695.7	845.8	0.085	814.8	0.010	850.8	0.205	716.6	0.944	799.9	109.5	13.7%
V_30_ (cc)	576.2	738.3	0.325	702.1	0.007	692.9	0.097	655.4	0.787	691.6	99.6	14.4%
V_35_ (cc)	465.5	629.1	0.142	589.8	0.007	595.8	0.088	495.7	0.827	566.3	91.8	16.2%
V_40_ (cc)	383.1	509.5	0.179	442.5	0.784	525.2	0.001	392.4	0.945	472.8	83.5	17.7%
V_45_ (cc)	301.2	419.7	0.164	408.7	0.008	458.8	0.274	397.5	0.422	375.9	76.6	20.4%
V_50_ (cc)	201.3	280.4	0.273	292.6	0.012	281.0	0.059	278.5	0.435	276.5	65.9	23.8%

The *P*_*1*_*, P*_*2*_*, P*_*3*_*,* and *P*_*4*_ represent the comparison between the 1-4 W and Plan respectively

### NTCP of small bowels

As shown in Table 2 (BL), the NTCP_C_ and NTCP_A_ based plan of pre-treatment were 2.0 and 59.2%, the shift% during treatment were 46.1 and 14.0% respectively. The difference of NTCP_A_ in 2 W and 4 W, and difference of NTCP_C_ in 2–3 W were significant compared with the plan pre-treatment (*p* < 0.05). As shown in Table 4, NTCP in supine patients were mildly larger than in prone patients, NTCP_C_ 4.9% vs 2.3% (*p* = 0.055) and NTCP_A_ 58.3% vs 55.7% (*p* = 0.109).

**Table 4:** Comparison of the small bowel dose-volume and NTCP between prone and supine patients

Variable	Supine position	Prone position	T	*p*
V_5_ (cc)	361.0 ± 113.2	208.0 ± 62.0	3.73	0.003
V_10_ (cc)	262.4 ± 78.5	191.4 ± 58.2	3.64	0.003
V_15_ (cc)	176.6 ± 47.2	160.0 ± 51.1	1.84	0.092
V_20_ (cc)	139.2 ± 44.7	134.7 ± 49.6	0.24	0.811
V_25_ (cc)	112.8 ± 41.9	110.3 ± 45.0	−0.28	0.777
V_30_ (cc)	86.8 ± 38.6	76.6 ± 37.9	−0.28	0.779
V_35_ (cc)	67.5 ± 34.1	60.3 ± 32.4	−0.02	0.980
V_40_ (cc)	52.4 ± 29.2	47.5 ± 28.9	0.06	0.953
V_45_ (cc)	38.8 ± 24.7	36.6 ± 24.4	0.18	0.856
V_50_ (cc)	21.3 ± 20.4	23.0 ± 16.8	0.24	0.813
D_max_ (cGy)	5341 ± 28	5341 ± 29	0.01	0.989
NTCP_C_ (%)	4.9 ± 2.9	2.3 ± 1.6	2.14	0.055
NTCP_A_ (%)	58.3 ± 7.1	55.7 ± 9.8	1.74	0.109

### Safety assessment of small bowels during treatment

**Fig. 4:**
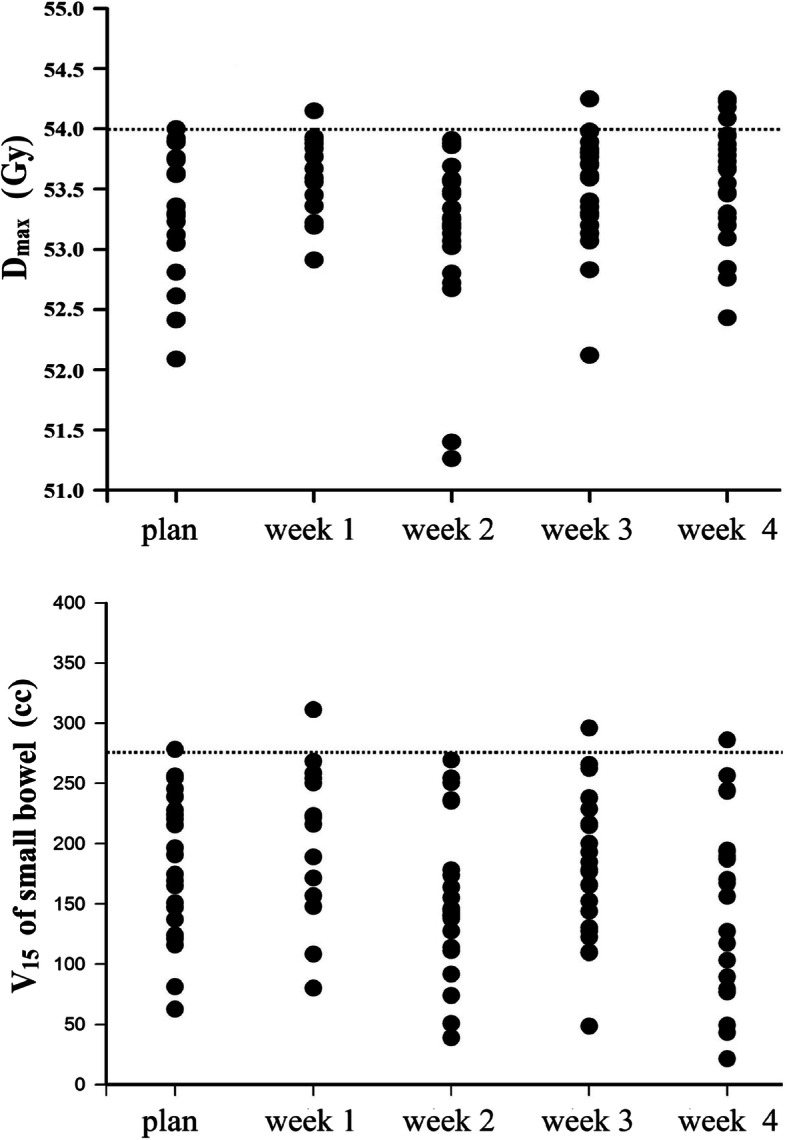
Safety assessment of the small bowel in 24 patients with rectal cancer during treatment. The top and bottom pictures are the D_max_ and V_15_ estimation, respectively

As shown in Fig. 4, V_15_ of the small bowel exceeded 275 cc 3 times (3/109) during treatment, with a maximum of 311.3 cc (over 13.18%). D_max_ of the small bowel > 54 Gy 5 times, and the maximum value was 54.3 Gy.

## Discussion

Because the small bowel is a radiosensitive organ, acute and chronic side effects may occur during rectal cancer radiotherapy. The side effects can be reduced by limiting the dose volume. However, evaluating the small bowel dose volume can be challenging. Characteristics of small bowel movement may weaken the dose-limiting function. The small bowel loops do not remain in the same positions at all times. They experience both oscillating displacements of the wall due to peristalsis and large amplitude shifts due to changes in content. The frequency of peristalsis can reach 8–11 times per minute, and it can combine into complex forms of motion at different times and spaces [26]. Small bowel movements have to be taken into account when evaluating the dose volume by contouring the BL, while the peritoneal space can account for any potential region that may be occupied by the small bowel and covering its movements, so it replaces the BL for dose constraints with clinical significance.

In this study, we first evaluated small bowel movement during treatment. Our results showed that variations of all BL’s dose-volume were larger than 28%, while most of PS were below 20% (V_5–40_), and variations in prone position was significantly lower than in the supine position (Fig. 2). Kvinnsland et al. studied the dose volume changes in the small bowel through 6 to 8 repeated CT scans in 10 patients with bladder cancer. Their results showed that the relative standard deviations of V_30.8_, V_49.5_, and V_53.5_ were 20, 24, and 26% respectively. The authors believed that small bowel dose limitations should be carefully considered when variations in the irradiation volume exceeded 20% [27]. Sanguineti et al. confirmed small bowel movement during prostate cancer radiotherapy by continuous CT scanning. The results showed that 280 cc of the small bowel completely changed position on planned CT, while only 20% remained in its original position [28].

The movement characteristics of the small bowel make it necessary to explore the reliability of the PS dose limit method for small bowel sparing in IMRT. We used V_15_ < 275 cc and D_max_ ≦ 54Gy as the safety standard for small bowel during treatment, our results showed that majority of D_max_ and V_15_ were meet the safety standard, and indicating that the PS limit method was feasible for small bowel sparing.

Although the recommended dose constraint from Robyn B was used in this study [16], there are slightly different research methods and irradiation techniques between the two. The PS dose and small bowel with PTV 45 Gy followed by tumour 5.4 Gy boost in the literature may be lower than the present study (50 Gy PTV dose), while the four-field conformal technique may lead to a higher dose than the IMRT technique used in this study. V_15_ < 830 cc used as the dose constraint in this study was relatively strict, approximately half of the plans (13/24) exceeded this standard, and the median value exceeded 10.71%. But even so, our results showed that the small bowel dose-volume could be further reduced by strictly limiting the PS dose, so it is appropriate to use V_15_ < 830 cc as the dose constraint.

Patients with prior abdominal surgery are tend to experience greater rates of radiation-induced enteritis [29], it may also affect the movement of small bowel during treatment. Because neoadjuvant treatment was not fully popularized in our hospital in 2014 and 2015, only 8 patients with neoadjuvant radiotherapy were involved in our study. Among the 8 patients, 3 were supine and 5 were prone position, the mixing of position effects make it difficult to compare the difference of small bowel movement between neoadjuvant and adjuvant radiotherapy patients.

Regarding the upper boundary of the PS and BL, Robyn B defined 1.5 cm above the PTV [16] while our study used RTOG of 1.0 cm [17]. There was no substantial difference between 1.0 cm and 1.5 cm because coplanar IMRT technology and absolute volume (cc) evaluation were used in this study. An upper boundary larger than 1 cm above the PTV should be adopted when using non-coplanar irradiation, while 2–5 cm should be used for tomotherapy [17].

The supine and prone position with a belly board are common therapeutic positions in IMRT for rectal cancer. Our results showed that dose-volume, NTCP and their variations of small bowel were less in prone than supine position (Fig.2 and Table 4), consistent with previous studies [30–33]. Nevertheless, the design reproducibility and target dose coverage were significantly superior in the supine position. Some studies reported that patient positioning in RT for rectal cancer patients may therefore be selected based on other factors such as the most comfortable position for the patients [33, 34].

The PS defined in this study included the small bowel, colon, and space between the intestines. The PS used objectively in IMRT planning can reduce the overall PS dose volume, making it easier to reduce the small bowel dose. It reduces high dose irradiation caused by small intestinal movement during treatment, so it has an advantage over the BL limit. Which uses only the small bowel as the objective function. Further research showed that there was a significant correlation of V_15_ between the PS and BL (Fig. 3, R = 0.455, *p* = 0.000), indicating that the PS can replace the BL as the objective function of the dose constraint in IMRT planning. However, when using the PS limit, attention should be paid to the occurrence of PS dose hotspots in the absence of BL evaluation, especially when the dose limits are more stringent, and dose hotspots in PS must be evaluated and avoided to prevent excessive small bowel irradiation.

Our study may be too broad in showing the amplitude of small bowel movement, because involving neoadjuvant and adjuvant therapy patients, which may be a limitation in our study. On the other hand, V_15_ as the primary dose-volume evaluation methods in this study was from conformal radiotherapy era, whether it is suitable for IMRT needs further clinical verification. Recent research shows that the moderate to high dose (V_20–40_) trends toward being significantly associated with acute toxity of small bowel in IMRT [35, 36].

## Conclusions

Our findings demonstrated that small bowel motion may lead to uncertainties in its dose volume and NTCP assessment during IMRT for rectal cancer. The BL movements were significantly greater than the PS and significantly less in the prone position than in the supine position. It is feasible to use the PS instead of the BL limit to spare the small bowel. V_15_ < 830 cc can be used as the dose constraint standard.

## Data Availability

The datasets used and analyzed during the current study are available from the corresponding author on reasonable request.

## Abbreviations

PS: Peritoneal space
BL: Bowel loops
IMRT: Intensity-modulated radiotherapy
NTCP: Normal tissue complication probability
QUANTEC: Quantitative Analysis of Normal Tissue Effects in the Clinic
CT: Computed Tomography
RTOG: Radiation Therapy Oncology Group
PTV: Planning target volume
MLCs: Multi-leave collimators
NCCN: The National Comprehensive Cancer Network
CTV: Clinical target volume
D_max_: Maximum dose
NTCP_C_: Chronic complication probability of the small bowel
NTCP_A_: Acute complication probability of the small bowel
V_30.8_, V_49.5_, V_53.5_: Volume receiving at least 30.8Gy, 49.5Gy, 53.5Gy
V_10_, V_15_, V_30_: Volume receiving at least 10Gy, 15Gy, 30Gy
